# Enhancing text-level reading fluency and engagement in immigrant children through a structured singing-based intervention

**DOI:** 10.3389/fpsyg.2025.1677981

**Published:** 2025-10-24

**Authors:** Eunjin Choe, Mihye Lee, Soo Ji Kim

**Affiliations:** ^1^Department of Music Therapy Education, Graduate School of Education, Ewha Womans University, Seoul, Republic of Korea; ^2^Department of Korean Education as a Foreign Language, Graduate School of Education, Ewha Womans University, Seoul, Republic of Korea

**Keywords:** reading fluency, reading attitude, immigrant children, singing-based intervention, music and language

## Abstract

**Introduction:**

Reading fluency is a key component of academic success, yet immigrant children often face difficulties in developing this skill in a new language due to limited phonological awareness, reduced exposure, and differences between home and school language environments. This pilot study examined the effects of a singing-based intervention designed to improve reading fluency and attitudes toward reading among immigrant children.

**Methods:**

Eight elementary school children from immigrant backgrounds participated in an eight-session program conducted twice weekly for 50 minutes per session. The intervention integrated rhythm-based word chanting, sentence singing, and songwriting. Pre- and post-intervention assessments included the Reading Fluency Test measuring accuracy, automaticity, and prosody, and a modified Reading Attitude Inventory assessing interest, habits, and perceptions. Semi-structured interviews were also conducted to explore participants’ experiences and perceived changes.

**Results:**

Quantitative analyses indicated overall improvements in reading fluency, particularly in accuracy and automaticity. Participants also reported increased reading interest and more positive literacy attitudes. Children with longer residence in Korea showed greater gains, though progress was also observed among those with limited exposure.

**Discussion:**

Findings provide preliminary evidence that singing-based interventions may enhance reading fluency and engagement among immigrant children. The rhythmic and melodic features of singing may support phonological and prosodic processing that underlie fluent reading. However, the small sample size, brief duration, and absence of standardized comprehension measures highlight the need for larger, longitudinal studies.

## 1 Introduction

Children from immigrant backgrounds represent a growing segment of the school population in many countries, including South Korea, due to trends in transnational marriage and labor migration (Maioli et al., 2021). These children often attend under-resourced schools and encounter language barriers, both of which contribute to lower academic achievement in reading, a domain requiring strong language proficiency (Mullis and Kelly, 2022).

Reading fluency—defined by automaticity, accuracy, and prosody—is essential for comprehension and academic success (Rasinski, 2012). Fluent reading enables students to allocate cognitive resources effectively toward meaning-making (Makebo et al., 2022). However, children from immigrant backgrounds in South Korea often face challenges in reading fluency due to limited exposure to Korean and differences between their home language and the primary language used in educational settings (Stolk et al., 2025).

Traditional fluency interventions often rely heavily on text repetition and phonics drills (Hudson et al., 2020). While effective for some children, these approaches may be difficult for children with limited language development or low motivation to engage with Franck and Delage (2022) and Mehigan (2020). In response to this issue, a new wave of studies highlights musical interventions—particularly singing—as potent tools to enhance phonological processing, prosodic awareness, and reading motivation (Descamps et al., 2025; Lewis and Kim, 2024; Panteleo et al., 2024).

Based on auditory-motor coupling and prosody facilitation theories, singing provides a multimodal context where rhythm, melody, and language are integrated. Previous research indicates that musical rhythm synchronizes neural clock mechanisms to enhance phonological decoding abilities, while melodic contours.

Recent systematic reviews indicate that music-based interventions can have positive effects on reading-related skills, though the evidence is not uniformly conclusive (Antonietti, 2022; Gordon et al., 2015). In particular, research involving multilingual immigrant learners has expanded over the past decade. For example, nursery song-based instruction was shown to significantly improve grammatical proficiency among newly arrived children—a skill considered foundational for fluent reading (Busse et al., 2018). Similarly, music participation has been found to support both cognitive and social adaptation in newly arrived immigrant students (Rinde and Kenny, 2021). Additionally, the value of rhythmic and melodic engagement in promoting literacy and affective outcomes among displaced youth has been underscored in recent systematic reviews of non-verbal arts interventions (Heynen et al., 2022).

Despite these promising findings, interventions to improve reading fluency among immigrant children remain understudied. The limited research that has been conducted has focused on vocabulary development or prosodic sensitivity rather than on fostering fluent reading across multiple text levels (Flaugnacco et al., 2015; Heynen et al., 2022; Kim and Petscher, 2016).

To contribute to this literature, this pilot study examined the effects of a structured singing-based intervention on reading fluency and reading attitudes of immigrant children. The intervention was designed to strengthen auditory-motor association and prosodic structuring and focused on stimulating children’s interest in and motivation for reading through the enjoyable musical activity of singing.

We addressed the following research questions:

1. Is there a difference in the pre- and post-reading fluency of immigrant children who participated in the singing intervention?
2. Is there a difference in the pre- and post-reading attitudes of immigrant children who participated in the singing intervention?
3. Following the intervention, how did the participating children describe changes, if any, in their reading experience?

## 2 Methods

### 2.1 Study design

This preliminary study employed a single-group pretest–posttest design to examine changes in reading fluency and attitudes toward reading among immigrant children who participated in a singing-based intervention. Quantitative data were collected through standardized assessment conducted before and after the intervention, while qualitative data were collected through interviews conducted after the intervention to explore participants’ subjective experience and perceived change.

### 2.2 Participants

Participants were eight elementary school students (ages 7–11 years) with immigrant backgrounds residing in Kyonggido, South Korea. Recruitment was conducted through an after-school education support center serving immigrant families. Following approval from the Ewha Womans University Institutional Review Board (IRB No. 202505–0014-01), eligible families were provided with study information and invited to give informed written consent.

Inclusion criteria were as follows:

- The parent had at least one child currently enrolled in a public elementary school in Kyonggido, South Korea.
- The parent, their spouse, or their child had to have immigrated to Korea after birth.
- The child had to possess sufficient Korean language proficiency to understand instructions and participate in activities, as determined through teacher reports and informal screening during the initial orientation session, and have no diagnosis of a reading disability.
- The parent had to voluntarily provide written parental consent and their participating child had to provide written assent.

Table 1 presents demographic and linguistic background information, including age, sex, parental nationality, year of arrival (if applicable), home and peer/school language use, and teacher-reported ratings of Korean communication and reading ability based on the International Standard Model of Korean Language Education (National Institute of the Korean Language, 2017).

**Table 1:** Participants’ demographic characteristics.

Pt	Age (in years)	Gender	Grade	parental nationality	Arrival in Korea (Korean exposure)	Home language	Language used with peers at school	Korean language proficiency	Korean reading ability
A	7	F	2	Both from Vietnamese	2024 (1 year)	Vietnamese	Korean Vietnamese	Moderate	Moderate
B	8	F	3	Both from Uzbekistan	2018 (7 years)	Russian	Korean	High	High
C	8	F	3	Russia (Father) Uzbekistan (Mother)	Born in Korea (8 years)	Russian	Korean	High	High
D	9	F	3	Both from China	2018 (7 years)	Korean	Korean	Moderate	Low
E	9	F	3	Both from China	2018 (7 years)	Korean	Korean	High	Moderate
F	10	M	4	Both from Russia	2024 (1 year)	Russian	Korean Russian	Low	Moderate
G	11	M	4	Both from Russia	2022 (3 years)	Russian	Korean Russian	Moderate	Moderate
H	10	M	4	Both from Uzbekistan	2022 (3 years)	Russian	Korean Russian	Moderate	Moderate

F, female; M, male; Pt, participant.

Korean communication ability was evaluated in terms of understanding both familiar and unfamiliar topics, conversing with teachers and peers at a natural rate and intonation, and expressing emotions and thoughts. One child born in Korea and three children who had resided in Korea for more than six years were rated at medium or high levels, while the four children who had resided in Korea for less than two years were rated at low to medium levels.

Reading ability was assessed by the ability to read sentences or short passages aloud with appropriate application of phonological and prosodic rules and to comprehend the content. Because it was not feasible to administer a formal standardized reading test, reading levels were determined based on teacher reports and the children’s utterances observed during the intervention. Where difficulties arose, children were allowed to read with teacher support, which was incorporated into the level ratings. Among the four children rated at medium or high levels in communication, three also showed medium or high levels of reading, but one child (Participant D) was rated at a low level. Although this child’s oral communication was fluent, the teacher reported slow comprehension, frequent inattentiveness, and nonresponsive behaviors in class. Reading assessments also revealed unclear word boundaries, syllable-by-syllable reading, repetition of the same segments, and reduced reading speed. This case illustrates the discrepancy that can occur between spoken communication and reading skills in immigrant children.

### 2.3 Intervention program

#### 2.3.1 Singing-based intervention

The singing-based intervention was implemented over eight group sessions, each lasting 50 min and occurring twice a week over a 4-week period. Each session was structured into three sequential phases—preparation, practice, and integration—to scaffold linguistic development through multimodal, music-based activities. The design of each phase was informed by both linguistic theory and research on auditory–motor coupling and prosodic facilitation. All sessions were held in a private room at the after-school education support center.

The preparation phase (15 min) focused on phonological awareness and word-level fluency. Children were introduced to several vocabulary items thematically aligned with the session content. The words to be used were selected based on the 2020 revised Korean National Elementary Language Curriculum and the language level of participants. During this phase, rhythmic chanting and simple body movements (e.g., clapping, stepping) were used to reinforce the syllabic structure and help pronounce target words. The music therapist provided beat-based cues to help the children clearly pronounce their target words and guided them to use the vocabulary in a story timed to the provided musical structure. Visual aids and group-based repetition were used to reinforce prosodic features such as stress and intonation. Previous studies have shown that rhythm-based word work enhances decoding efficiency and phonological processing, particularly for learners with limited exposure to the dominant language (Flaugnacco et al., 2015; Goswami, 2011; Patel, 2003).

The practice phase (15 min) targeted sentence-level prosody and spoken fluency. This phase emphasized singing sentences to promote natural spoken language development, using simple melodic phrases. In this phase, songs were sung with fill-in-the-blank or sentence-based lyrics to encourage longer and more natural reading since lyrics, rhythm, and melodic contour can aid expressive speech and support sentence recognition and auditory-motor integration. Research has shown that melodic prosody plays a role in improving expressive reading and fluency in multiple languages (Bonacina et al., 2021; Gordon et al., 2015; Tierney and Kraus, 2014).

The integration phase (20 min) aimed to improve reading rhythm by singing songs created with lyrics using the target words. This stage involved the children composing songs using the lyrics they had just written in the practice phase. Rhythm structures of 4, 8, and 12 bars were provided to help participants fill in the lyrics and naturally create songs. Ultimately, this process has been shown to lead to the acquisition of words and sentence structures (Busse et al., 2018). The song-making process gradually helps expand expression, and singing helps with spoken expression activities. The applications of musical tasks, including musical storytelling and songwriting, have been shown to improve vocabulary retention, reading motivation, and communicative confidence, particularly among linguistically marginalized learners (Busse et al., 2018; Rinde and Kenny, 2021).

**Figure 1:**
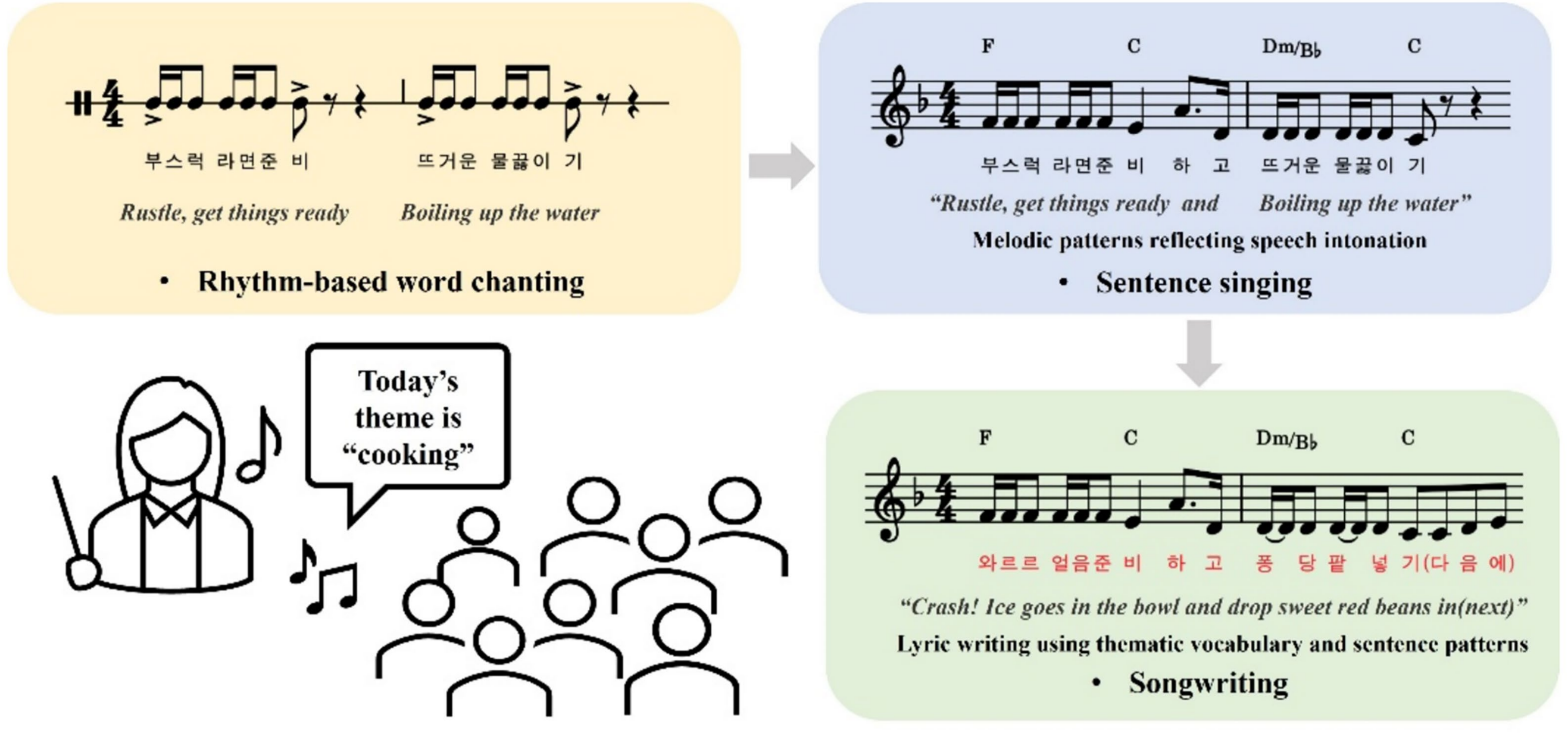
The stepwise singing-based intervention.

Through the above steps (see Figure 1), participants progressed from phoneme-level decoding to sentence formation and creative language use. Various elements such as rhythm, melody, and body movement were combined with reading tasks to maintain engagement and enhance language processing. All sessions were conducted in Korean.

#### 2.3.2 Session themes and vocabulary selection

The themes and vocabulary used in the singing-based sessions were taken from the 2020 revised Korean National Elementary Language Curriculum. Session topics were selected to reflect both narrative and expository text structures so as to achieve the specific goals of the intervention while maintaining continuity with regular school classes. Considering the diverse linguistic backgrounds of the participants, themes were based on everyday experiences commonly encountered by children in elementary school (see Figure 1). The vocabulary selection was adjusted in complexity to individual needs. It was simplified for participants at low and moderate levels of Korean reading ability and expanded slightly for participants rated as possessing high Korean reading ability. This differentiation was discussed with Korean teachers before the intervention began.

Regarding the vocabulary used in each session, researchers considered two sources: words embedded within the target texts for each theme and foundational vocabulary recommended by the Korea Institute for Curriculum and Evaluation (KICE). In the early sessions, the focus was on concrete, high-frequency nouns related to daily life—such as “family,” “school,” and “food”—and simple declarative sentences composed of fewer than three word phrases (e.g., “My sister likes ice cream,” “I pass by the flower shop”). In later sessions, the vocabulary scope expanded to include descriptive adjectives and ideophones to facilitate more nuanced emotional and situational expression.

Syntactic complexity was gradually increased by introducing compound and complex sentences using conjunctions (e.g., “Because we played instruments, we had fun” and “We got in the red fire truck to put out the fire”). Musical elements were deliberately used to reinforce targeted sentence structures. For example, descending melodic contours were used to indicate completeness at the end of statements, and repetition of rhythmic patterns was applied to the syllabic structure of each sentence to help participants internalize the boundaries between words and phrases. When introducing compound sentences, the resolution of pitch was delayed, transitions between clauses were indicated, and the syntactic endings were emphasized through leaps or changes in melody. These musical adjustments were not arbitrary; rather, they were meant to help develop syntactic awareness and prosodic sensitivity within the structured musical context.

### 2.4 Assessment tools

#### 2.4.1 Reading fluency

To measure the participants’ change in reading fluency, we extracted and used the word fluency and sentence fluency items from the Reading Fluency and Comprehension Test provided by the National Center for Basic Skills Support in Korea. The reading fluency test measures whether a given text is decoded accurately and fluently and measures accuracy, automaticity, and prosody. Each subscale score is calculated based on the number of correctly read words, the number of misreads, or prosody. A higher score indicates a more fluent reading. Subscale scores were interpreted using the KICE Reading Inventory (KRI) sentence reading fluency judgement criteria and subfactor scale criteria (Fuchs et al., 1982; Rasinski, 2004; Song, 2012). All readings were recorded and analyzed, and changes in reading fluency that appeared during the singing intervention were analyzed through video observation for each session in addition to voice evaluation using KRI.

Because the primary aim of this pilot study was to explore changes in fluency (accuracy, automaticity, prosody) over a short four-week intervention, we prioritized the word and sentence fluency indices of the standardized KICE Reading Inventory (KRI) (National Center for Basic Skills Support, 2023a). Standardized indices of reading comprehension were not included at the design stage, which we acknowledge as a limitation and address in the Discussion.

#### 2.4.2 Reading attitudes

Reading attitudes were measured using a modified version of the Reading Interest Scale (Mata, 2011; Song, 2015) and the Reading Habit and Perception Inventory (Lee, 2016; National Center for Basic Skills Support, 2023b). The Reading Interest Scale contains 16 items rated on a 4-point Likert scale (total score range: 16–64) and assessed enjoyment, perceived value, and reading self-efficacy. The Reading Habit and Perception Inventory contains 11 questions related to reading experiences in daily life, reading habits, genre preferences, and self-awareness of reading. To better capture children’s experiences and conceptions, the inventory was designed with both multiple-choice and open-response questions. Open-ended questions were analyzed qualitatively. During administration of the inventory, the researcher provided individual explanations as necessary to help children at lower levels of Korean reading ability respond to the questions.

#### 2.4.3 Post-intervention interviews

Upon the completion of the intervention, brief semi-structured interviews were conducted individually with each participant. The purpose was to gather additional perspectives on their experiences throughout the program and any perceived changes in their reading-related behaviors or attitudes. Interview prompts focused on the children’s level of engagement, emotional responses to the sessions, and self-reported shifts in motivation or reading habits. Conversations were held in a quiet, familiar setting to encourage open responses and minimize discomfort. Example prompts included, *“What was the most enjoyable part of the singing activities?,” “Did you find yourself listening to music or singing more often in daily life during the program?,”* and *“Have your feelings or behaviors around reading books or words changed since participating in the activities?”*

All interviews were conducted solely in Korean by the researcher, using a structured format in which the researcher posed questions and participants provided responses. Each interview was audio-recorded and transcribed verbatim in Korean, and all coding and analysis were completed in Korean to preserve linguistic nuances. For manuscript preparation, transcripts and analytic findings were translated into English, with back-translation procedures applied to ensure semantic accuracy.

The coding process followed Braun and Clarke’s (2006) guidelines: familiarization with the data, generation of initial codes, review of codes, and identification of themes. Two independent coders (one with training in music therapy and one in Korean language education) received standardized coder training with a manual and pilot exercises. They jointly coded approximately 20% of transcripts to refine a shared codebook (including code labels, operational definitions, inclusion/exclusion criteria, and sample excerpts). The full dataset was then double-coded, with discrepancies resolved through consensus meetings that included a third reviewer. Coding units were defined at the level of meaning segments, and rigor was enhanced through the maintenance of an audit trail and analytic memos. Intercoder reliability was calculated, yielding 91% percentage agreement. To protect confidentiality, pseudonyms were used in presenting excerpts.

#### 2.4.4 Fidelity and reliability

To ensure the fidelity of the intervention, two certified music therapists (KCMT) with over 6 years of experience and one elementary school teacher specializing in multicultural education reviewed the program for content validity. They rated the relevance and appropriateness of each session component using a 5-point Likert scale. The average content validity index across all items was above 0.85, indicating high program fidelity.

Interrater reliability for reading fluency scoring was calculated based on randomly selected pre- and post-test recordings. Agreement levels were 91% for word reading accuracy and 95–96% for sentence accuracy, and 83% for prosody, confirming acceptable reliability for this pilot study. A session-by-session fidelity checklist, participant attendance records, and exemplar intervention materials (lyric sheets, rhythm patterns, and song structures) are provided in the Supplementary material to support reproducibility.

## 3 Results

This section presents the quantitative and qualitative outcomes of the singing-based intervention. Pre- and post-test results for reading fluency and reading attitudes were analyzed using Wilcoxon signed-rank test, which is more robust with small sizes and non-normal distribution. Effect sizes were estimated with Cohen’s *d* for paired samples, and 95% confidence intervals were calculated wia bootstrapping. Interview responses were analyzed thematically to explore participants’ subjective experiences.

### 3.1 Participant characteristics

Table 1 presents the demographic and linguistic background information of the eight participants. Descriptive summaries indicated that increases in reading automaticity and prosody were observed across children of length of Korean exposure (including those with ≤3 years of residence), while gains in accuracy were more pronounced among those with initially lower baseline performance. Given the small sample, contextual variables were not used as covariates but are reported here to aid interpretation.

### 3.2 Changes in reading fluency

#### 3.2.1 Word reading fluency

Children’s word reading fluency increased significantly from pre-test (*M* = 0.79, SD = 0.13) to post-test (*M* = 0.92, SD = 0.07), *W* = 0, *p* = 0.17. The effect size was large, *d* = 1.77, 95% CI [1.02, 4.88]. All participants, except one who had already reached the ceiling score at baseline, demonstrated notable improvement. According to national reading benchmarks provided by the KICE, five participants initially scored at or below the acceptable threshold of 75%. Post-intervention results showed that all eight participants scored at or above 80%, which is considered adequate proficiency for the elementary grades (see Table 2).

**Table 2:** Pre-post results of word reading fluency.

Participant	Pre-test (%)	Post-test(%)	Change
A	60	80	+20
B	90	95	+5
C	90	100	+10
D	75	90	+15
E	100	100	+0
F	75	90	+15
G	75	90	+15
H	70	90	+20
M (SD)	79.3 (12.93)	91.8 (6.51)	+12.5

#### 3.2.2 Sentence reading fluency

Sentence reading fluency was assessed based on three factors: reading accuracy, automaticity, and prosody. Pretest and posttest changes were measured using two types of texts: narrative and explanatory. The participants’ pretest and posttest sentence reading fluency scores are as follows.

##### 3.2.2.1 Reading automaticity

Participants demonstrated significant improvement in sentence reading automaticity across both narrative and expository text types (see Table 3). For narrative passages, score increased from M = 135.0 (SD = 48.80) to M = 165.6 (SD = 50.65), W = 0, *p* = 0.08, *d* = 2.59, 95% CI [2.13, 4.35]. For expository passages, scores rose from *M* = 142.9 (SD = 48.56) to *M* = 178.0 (SD = 57.15), *W* = 0, *p* = 0.08, *d* = 2.57, 95% CI [2.10, 4.47]. Notably, Participants B, E, and G demonstrated substantial gains of more than 40 words per minute across both narrative and expository texts. Participant B, who had over seven years of Korean language exposure and was assessed as having a “high” level of Korean proficiency and reading ability, maintained consistently high performance and showed further improvement. Participant E, despite also having over seven years of Korean exposure, began with a lower baseline than B but demonstrated a marked gain of 41 words in both text types. Participant G, evaluated as having a “moderate” level of Korean proficiency and only three years of exposure, exhibited the largest overall gain. These findings suggest that improvements in reading automaticity occurred regardless of participants’ initial reading proficiency or duration of Korean language exposure. No consistent relationship was observed between the magnitude of improvement and participants’ language background (see Table 4).

**Table 3:** Sentence reading automaticity: pre-post comparison for narrative and expository texts.

Participant	Narrative text			Expository text		
	Pre-test (n)	Post-test (n)	Change	Pre-test (n)	Post-test (n)	Change
A	109	128	+19	98	117	+19
B	224	262	+38	233	282	+49
C	183	205	+22	166	193	+27
D	70	93	+23	75	100	+25
E	125	166	+41	135	176	+41
F	149	166	+17	173	215	+42
G	110	159	+49	131	187	+56
H	110	146	+36	132	154	+22
M (SD)	135.0 (48.80)	165.6 (50.65)	+30.6	142.8 (48.56)	178.0 (57.16)	+35.2

**Table 4:** Sentence reading accuracy: pre-post comparison for narrative and expository texts.

Participant	Narrative text			Expository text		
	Pre-test (%)	Post-test (%)	Change	Pre-test (%)	Post-test (%)	Change
A	97.3	96	−1.3	97	98.3	+1.3
B	98.6	99.2	+0.6	98.3	98.6	+0.3
C	96.8	96.2	−0.6	97.6	98.4	+0.8
D	92.1	94.9	+2.8	89.2	96.1	+6.9
E	98.4	97.6	−0.8	98.5	100	+1.5
F	93.7	94.3	+0.6	94	97.2	+3.2
G	98.2	99.3	+1.1	94.9	96.3	+1.4
H	98.2	98.6	+0.4	93.6	97.4	+3.8
M (SD)	96.6 (2.43)	97 (1.94)	+0.4	95.4 (3.15)	97.7 (1.29)	+2.3

##### 3.2.2.2 Reading accuracy

Sentence reading accuracy showed no significant changes for narrative texts (pre *M* = 96.6%, SD = 2.43; post *M* = 97.0%, SD = 1.94; *p* > 0.05), but significant gains were observed for expository texts (pre *M* = 95.4%, SD = 3.15; post *M* = 97.7%, SD = 1.29), *W* = 0, *p* = 0.16, *d* = 1.2, 95% CI [0.4, 2.3].

Among all participants, only Participant D demonstrated improvement in both text types, with accuracy gains of 2.8% for narrative and 6.9% for expository passages. This suggests a substantial enhancement in decoding precision. Participants F and H also showed meaningful improvements in expository texts, with increases of 3.2 and 3.8%, respectively. In contrast, children who exhibited high initial accuracy rates (above 97%) tended to maintain their performance levels with minimal change. These findings indicate that reading accuracy improvement was not clearly associated with the duration of Korean language exposure. However, greater improvements were observed among participants with lower baseline accuracy, suggesting that the structured singing-based intervention—through repeated and rhythmically supported exposure—may have contributed to enhanced decoding accuracy, particularly for those with initially limited proficiency.

##### 3.2.2.3 Reading prosody

Prosody scores increased significantly across both text types (see Table 5). For narrative passages, the mean score increased from 9.5 (SD = 3.50) to 12.6 (SD = 2.44), *W* = 0, *p* = 0.08, *d* = 2.30, 95% CI [1.58, 4.65]. For expository passages, scores increased from 9.9 (SD = 3.27) to 12.5 (SD = 2.26), *W* = 0, *p* = 0.08, *d* = 2.02, 95% CI [1.51, 3.68]. Participants with low initial prosody scores (≤ 8) demonstrated the most notable improvement during the intervention, while participants who had lived in Korea longer increased high prosody performance. Notably, participant D, who had lived in Korea for 7 years, showed significantly lower prosody from the beginning and displayed a distinct reading pattern characterized by syllable-by-syllable segmentation and noticeably slow speed. This was a pattern observed in other participants who had lived in Korea for 1 to 3 years, suggesting that D’s prosodic delay may have been influenced by individual differences in language acquisition and reading development.

**Table 5:** Sentence reading prosody: pre-post comparison for narrative and expository texts.

Participant	Narrative text			Expository text		
	Pre-test (score)	Post-test (score)	Change	Pre-test (score)	Post-test (score)	Change
A	8	12	+4	8	12	+4
B	15	16	+1	14	15	+1
C	13	15	+2	13	15	+2
D	4	8	+4	4	8	+4
E	11	14	+3	12	14	+2
F	10	12	+2	11	12	+1
G	7	12	+5	9	12	+3
H	8	12	+4	8	12	+4
M (SD)	9.5(3.50)	12.6(2.44)	+3.1	9.8(3.27)	12.5(2.26)	+2.7

### 3.3 Changes in reading attitudes

#### 3.3.1 Reading interest

Participants’ overall reading interest showed a statistically significant improvement following the intervention (see Table 6). Scores improved significantly from pre-test (*M* = 46.6, SD = 8.21) to post-test (M = 51.2, SD = 6.04), *W* = 3, *p* = 0.47. The effect size was moderate, *d* = 0.85, 95% CI [0.2, 1.9]. Subscale analysis revealed variation based on participants’ initial interest levels. Participants who initially reported relatively high levels of reading interest (e.g., C, E, F, and H) showed limited change, with some maintaining their scores and others exhibiting slight declines. In contrast, Participant D, who had the lowest initial interest, demonstrated an increase specifically in the “reading enjoyment” domain. Participant B, whose baseline interest was moderate, showed improvement across all three subscales, representing the most substantial overall gain. Similarly, Participant G displayed balanced increases across all domains, reflecting a general enhancement in reading interest. These findings suggest that the intervention was particularly effective for children with initially low to moderate levels of reading interest, regardless of their duration of Korean language exposure. The observed variability highlights the influence of individual characteristics—such as personal disposition, language environment, and learning attitudes—on the responsiveness to the intervention.

**Table 6:** Pre-post results of reading interest.

Participant	Reading enjoyment		Perceived value and importance of reading		Reading self-efficacy		Total score		Change
	Pre	Post	Pre	Post	Pre	Post	Pre	Post	
A	24	26	5	13	18	17	47	56	+9
B	18	22	10	13	14	20	42	55	+13
C	26	26	12	12	16	16	54	54	0
D	7	14	10	10	15	16	32	40	+8
E	25	25	14	14	20	20	59	59	0
F	22	21	11	13	16	15	49	49	0
G	18	20	9	14	15	16	42	50	+8
H	22	22	11	11	15	14	48	47	−1
M (SD)							46.6 (8.21)	51.2 (6.04)	+4.6

#### 3.3.2 Reading habit and perception

Overall, positive changes were observed in children’s responses regarding their reading practices, reading experience, self-confidence, and awareness of the need for reading. For example, Participant A expressed the need for Korean language book reading and the will to read voluntarily, and participant D, whose initial reading level was low, responded, “I do not need to read Korean language books” in the pretest, but responded, “I want to read the books because they are fun” in the posttest, showing a change in reading habit. In the case of children who were rated as possessing high Korean Language Proficiency (Participants B, C, E), they consistently demonstrated positive attitudes at pretest and posttest, indicating specific recognition of the value of reading.

In contrast, Participants G and H, both of whom had been exposed to Korean for approximately three years and were rated as having “moderate” reading ability, initially responded “I do not know” to many of the pretest items, reflecting uncertainty or lack of confidence. However, by the posttest, Participant G stated, “I want to express my thoughts better by improving my Korean,” and Participant H shared, “Reading is difficult and takes a lot of time, but I still want to learn,” demonstrating emerging awareness of the need for reading. Participant F, who had the shortest duration of Korean exposure, initially reported reluctance to read Korean books and expressed difficulty with Korean reading. Nonetheless, post-intervention responses such as “I think I can get better at Korean if I read Korean books” and “I want to read all kinds of books like storybooks and picture books” revealed a positive shift in reading perception.

### 3.4 Observed changes in prosody and rhythm during the intervention

Participants showed various changes depending on their exposure to Korean and their initial language level. Participant A, who had been exposed to Korean for a short period of time, had no difficulty in daily communication, but initially had difficulty singing due to low voice intensity, monotonous intonation, and errors in consonants and double consonant processing.

Participants B, C, and E, who had no difficulty using Korean, actively and proactively participated in reading and singing activities from the beginning of the intervention and maintained stable performance overall. Among them, Participant C was initially reluctant to participate in singing due to low voice intensity and lack of confidence, but halfway through the intervention, her voice intensity and spontaneity improved, and she was able to reflect the prosody of each word, showing clearer speed, volume, and rhythm expression.

Meanwhile, Participant D showed difficulty in reading and singing despite a relatively long exposure period to Korean, and slow speed, distracted attitude, and avoidant behavior were observed in the early stage of the intervention. However, from the middle stage of the intervention, gradual changes in participation were observed, with attempts at singing phrases as well as questions and practice attempts for self-expression.

Participants F, G, and H, who predominantly used their native languages in daily life, initially demonstrated low engagement and passive attitudes during the intervention. Participant F, who had limited exposure to Korean and faced challenges in Korean communication, exhibited features such as sentence omission and monotonous intonation in the early stages. However, in the latter half of the intervention, increased emotional expression and engagement were observed during group singing activities, along with improvements in reading fluency marked by more stable pacing and spacing.

Participants G and H showed initial characteristics such as repetition of the same expression in the rhythmic changing, difficulty in processing consonants, and low vocal intensity, but with repeated musical stimulation, they were able to compose lyrics using new vocabulary. In the case of H, stabilization of performance was confirmed toward the end of the intervention, such as increasing spontaneous singing participation, reading by breaking up sentences, and reduced hesitation.

### 3.5 Qualitative insights from participant interviews

Post-intervention interviews provided additional perspectives on how participants engaged with the singing-based program. Analysis of the transcripts yielded acceptable intercoder reliability (91% agreement; see Method 2.4.4), and several recurring themes were identified across participants, including enjoyment, increased interest in reading, and ease in using Korean during musical activities.

#### 3.5.1 Enjoyment and engagement

Six out of the eight participants reported that they enjoyed the singing and songwriting activities. Three children specifically noted that reading became more interesting when integrated with music. Participant D, who had initially demonstrated low reading proficiency, explained that “the melodies stayed in my head even after class,” suggesting that musical memory supported ongoing engagement. Participant B, who was already proficient in Korean reading, reflected, “I did not like singing before, but now I enjoy it,” indicating that the intervention fostered new motivation even among children with strong baseline skills. In addition, children who primarily used their native language at home and initially reported low reading motivation remarked that the singing activities made reading feel easier and more enjoyable. For instance, Participant commented, “reading is hard, but when we sing, it feels less difficult,” and participant noted, “I wanted to read more because the songs were fun.” These illustrative quotations provide further evidence that integrating music into reading tasks enhanced both emotional engagement and willingness to participate.

These responses suggest that the musical components of the program contributed not only to increased engagement but also to a shift in participants’ emotional and cognitive orientation toward reading, particularly among those who initially showed low motivation or struggled with Korean literacy.

#### 3.5.2 Language use and expression

Among participants who predominantly used their native language at home and school, several reported increased use of Korean during communication and task performance within the intervention setting. These children began to produce longer and more detailed sentences, indicating a positive shift in their expressive use of Korean. For example, Participant shared, “When I made songs, I could speak in longer sentences than before,” while Participant noted, “At first I only used short words, but later I wanted to say more things in Korean.” Such comments illustrate a gradual expansion of expressive language competence.

## 4 Discussion

This pilot study explored the potential effects of a singing-based intervention on the reading abilities of immigrant children. The intervention was designed based on the structural similarities between music and language, incorporated rhythm-based chanting, melodic sentence singing, and narrative songwriting with performance. To evaluate pre- and post-intervention changes, data were collected focusing on reading fluency and reading attitudes, including reading interest, reading habits, and reading perceptions. Formal correction procedures (e.g., Holm–Bonferroni) were not applied, as this was an exploratory pilot study with a small sample size. Instead, we emphasized effect sizes and confidence intervals to aid interpretation. Additionally, individual semistructured interviews were conducted after the final session to capture qualitative changes associated with participation in the singing-based intervention. The main findings and their implications are discussed next.

Reading fluency improved across participants, with notable gains in automaticity and prosody. All eight participants scored above the grade average in word reading fluency, and substantial improvements were observed in sentence reading fluency, particularly in automaticity and prosodic features. These results may be partly attributed to the repetitive exposure to target vocabulary introduced through rhythm-based chanting and reinforced through melodic sentence singing. Such repetition appears to have supported participants’ awareness of sentence structure and contributed to faster processing. These findings align with previous studies suggesting that rhythm- and melody-based repetition can facilitate word recognition and syntactic processing (Chow and Brown, 2018; Merrill et al., 2012).

Differences between text types were observed, as improvements in sentence reading fluency were more pronounced in expository texts than in narrative passages. Because expository reading typlically requires higher cognitive and linguistic processing, these results may reflect the impact of structured, repetitive singing activities on phonological awareness and syntactic integration. This Such findings are consistent with prior research indicating that auditory rhythm patterns can facilitate phonological and syntactic integration (Angel and Sideroff, 2024; Lewis and Kim, 2024).

Reading attitudes and motivation appeared to benefit from the stepwise design of the intervention. Immigrant children often struggle with oral expression due to differences between their home language and the language used at school and differences in reading ability related to their sociolinguistic backgrounds. To address this, the program was structured to gradually expand from the word level to the sentence level and then to the narrative level and included collaborative music-making activities. This group-based format fostered psychological bonding and stability, reducing the cognitive burden associated with oral expression and promoting active participation. This study’s findings support earlier studies suggesting that stepwise music-based activities may contribute to emotional stability and promote language use among children with low academic achievement (Chen et al., 2024; Cores-Bilbao et al., 2019). This pilot study found that participants who initially showed signs of tension or avoidance during singing activities demonstrated a clear change in their participation attitude by the end of the intervention, such as voluntarily raising their hands to suggest ideas for group tasks.

Expressive language development was supported through the narrative component, which emphasized self-expression. Building on earlier findings that expressive music-making can encourage spontaneous language use (Hallam, 2019), the sessions provided opportunities for children to embed newly acquired vocabulary and structures into melodic contexts. This appeared to support intonation, phrasing, and emotional expression in naturalistic ways (Scherer et al., 2015). Children with limited Korean exposure gradually expanded their vocabulary, produced connected sentences, and demonstrated greater vocal intensity and sentence completion. In some cases, connectives and grammatical adverbs were also carried over into written tasks, and teachers observed increased Korean use in daily communication. Notably, Participant D, who had fluent oral communication but weak reading skills at baseline, showed partial gains in prosody and sentence completion, suggesting that structured singing may help narrow the common gap between spoken communication and reading among immigrant children. These results echo prior work showing that combining singing and speaking can facilitate grammar acquisition and broader language-related cognitive skills (Busse et al., 2018; Golinkoff and Hirsh-Pasek, 2008; Joo et al., 2024).

Taken together, the results indicate that the singing-based intervention supported improvements in phonological awareness, reading fluency, and expressive competence. Rhythm and melody appeared to promote both linguistic and behavioral changes, offering benefits beyond static text-based approaches. Narrative song formats, in particular, helped children engage more meaningfully with text and use Korean with greater fluency. These outcomes suggest that singing-based interventions hold promise as an educational strategy for enhancing literacy development in immigrant children.

Several limitations should be noted. The small sample size, short eight-session duration, and heterogeneous participant characteristics limit generalizability and render the findings preliminary. Factors such as length of residence in Korea, home language use, and prior literacy experience may also have shaped outcomes but could not be fully disentangled within this design. In addition, although standardized fluency measures were employed, no formal test of reading comprehension was included, and socioeconomic indicators (e.g., parental education, occupation, home learning support) were not collected. These omissions restrict interpretation and leave open the possibility of residual confounding.

Future research is needed to address these limitations. Larger and more homogeneous samples, longer-term interventions with follow-up evaluations, and the inclusion of comprehension, vocabulary, and socioeconomic measures will help strengthen external validity. Overall, this study should be regarded as preliminary evidence: although definitive conclusions cannot yet be drawn, the findings provide important initial insights into the potential of singing-based interventions to support reading development in immigrant children and lay a foundation for more rigorous educational and clinical applications.

## Data Availability

The original contributions presented in the study are included in the article and the Supplementary material. Further inquiries can be directed to the corresponding author, Soo Ji Kim (specare@ewha.ac.kr).
