# Supplementary material for: Enhancing text-level reading fluency and engagement in immigrant children through a structured singing-based intervention
Source: Front Psychol. 2025 Oct 24;16:1677981. doi: 10.3389/fpsyg.2025.1677981 (PMC12593454; doi:10.3389/fpsyg.2025.1677981)
Supplement: Supplementary file 1 [file Supplementary_file_1.pdf]

Supplementary Material 1. Session Fidelity Checklist

Fidelity Assessment

The fidelity of the singing-based intervention was assessed by a music therapist holding a master's degree. Randomly selected video recordings of intervention sessions were reviewed, and a 5-item, 5-point Likert scale checklist was used to evaluate adherence. Fidelity scores were converted into percentages according to the following formula:

$$\text{Fidelity} = \frac{\text{Observed intervention components}}{\text{Planned intervention components}} \times 100$$

The mean fidelity score across sessions was **96%**, indicating high adherence to the intervention protocol.

Fidelity checklist for singing-based intervention

Each item was rated on a 5-point likert scale (1= strongly disagree, 5 = strongly agree). Two independent raters completed the checklist after each session.

Table S1. Fidelity checklist for singing-based intervention

Item	Content	Rating Scale (1-5)
	The session objectives were clearly delivered and consistent with the intervention manual.	1 2 3 4 5
2	Target vocabulary and tasks were implemented as specified.	1 2 3 4 5
3	The rhythm- and singing-based activities were conducted in accordance with the session plan.	1 2 3 4 5
4	Participants actively engaged in the structured activities with appropriate guidance.	1 2 3 4 5
5	The session was implemented with high overall adherence to the intended structure.	1 2 3 4 5

Supplementary Material 2. Participant Attendance and Adherence

Attendance Rates

The overall mean attendance rate across sessions was **81.25%**. Table S1 presents session-by-session attendance.

Table S2. Session-by-session attendance record

	1st	2nd	3rd	4th	5th	6th	7th	8th
A	O	O	O	O	O	X	O	O
B	X	O	O	X	O	X	O	X
C	X	O	O	X	O	O	O	O
D	O	O	O	O	O	O	O	O
E	O	O	O	O	O	O	O	O
F	O	O	O	O	O	O	O	O
G	O	O	O	O	O	X	X	O
H	O	O	X	X	O	X	O	O

Supplementary Material 3. Exemplar intervention materials

Lyric sheet and rhythm structure (Session 5-6 example: “Today I’m the Chef”)

Korean Lyrics:
오늘은 내가 요리사 예예예
만들자 만들자 라면을 만들자
부스럭 라면 준비하고 보글 물 끓이기 (다음에)
쏙쏙 스프 넣으면 완성이다
랄랄라 랄라 라라랄라 침이 꼴깍 예!

English Translation:
Today I'm the chef, yeah yeah yeah
Let's make it, let's make it, let's make ramen
Rustle, get things ready and boiling up the water (next)
Add the soup and it’s done!
La la la la la la la la la, my mouth waters, yeah!

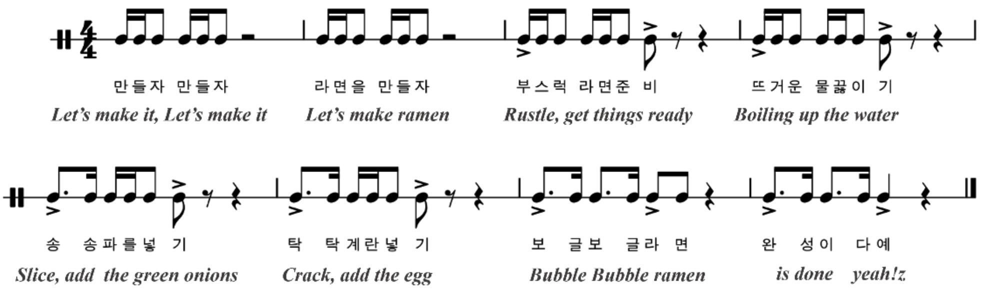

Figure S1. Lyric and rhythm structure
